# Global climate change: The dangers of heatwaves for chronic obstructive pulmonary disease patients cannot be ignored

**DOI:** 10.7189/jogh.14.03032

**Published:** 2024-09-06

**Authors:** Zhenggang Zhu, Tingting Deng, Xiaoyan Pan

**Affiliations:** 1 School of Nursing, Hunan University of Chinese Medicine, Changsha, China; 2 School of Nursing, Wenzhou Medicine University, Chashan Town, Wenzhou, China; 3 School of Health Sciences, Universiti Sains Malaysia, Kubang Kerian, Malaysia

Due to global climate changes, extreme weather events such as heatwaves, droughts, wildfires, hurricanes, snowstorms, and floods are becoming more frequent worldwide [1]. Especially with global warming, heatwaves have become more frequent, intense, and longer lasting in the 21st century [2]. In 2022, record-setting heatwaves were recorded in the northern hemisphere and affected areas and populations on an unprecedented scale. Chronic obstructive pulmonary disease (COPD) is a long-term inflammatory condition characterised by airflow limitation and ongoing respiratory symptoms, including shortness of breath, coughing, excessive mucus in the airways, and wheezing [3]. COPD is recognised as an environmental disease that is directly attributable to numerous environmental factors, such as climate change, cigarette smoking, and air pollution [4]. Higher temperatures, especially during summer heatwaves, can cause COPD exacerbations [5]. Climate change is predicted to increase the frequency of extreme weather events, such as heatwaves, which can exacerbate air pollution levels and further exacerbate COPD [6].

The health problems caused by heatwaves are often less obvious than those caused by meteorological disasters such as floods and hurricanes. Its impact on the human body is a cumulative process, especially for patients with chronic cardiovascular and respiratory diseases such as hypertension, heart disease, COPD, and asthma [7]. There is a delayed effect of five days or even longer of a heatwave; namely, although the temperature returns to normal after the heatwave, the adverse health effects of the heatwave, such as aggravation of diseases or even death, continue for more than five days [8]. Such a delayed effect makes it difficult to assess the health impacts of heatwaves and makes the public less aware of the health risks of heatwaves. Currently, heatwaves have been studied more in cardiovascular diseases. Limited studies of heatwaves have been conducted on respiratory diseases such as COPD [9]. However, the dangers of heatwaves for COPD patients should not be overlooked.

## CAUSES OF FREQUENT GLOBAL HEATWAVES

In general, a heatwave is a period of excessively hot weather that people cannot adapt to [10]. Due to global variations in climate factors, such as topography, atmospheric circulation, humidity, and people’s physiological adaptations to climate change, it is not easy to standardise the definition of a heatwave globally because different regions adopt different thresholds [11]. For instance, the World Meteorological Organization defines a heatwave as five or more consecutive days where the daily maximum temperature exceeds the average maximum temperature by 5°C (9°F) [10]. China calls the process of high-temperature weather with daily maximum temperature reaching or exceeding 35°C for more than three consecutive days as the heatwave [12]. There is no globally standardised definition of a heatwave or standardised criteria for measuring the effects of heatwaves on human health. There may be errors in assessing heatwave hazards due to differences in heatwave assessment systems in different regions. Although meteorological services define heatwaves in terms of temperature, humidity is also an important factor that affects human perception. Depending on the humidity, heatwaves can be categorised as dry or humid [13]. Both dry and humid heatwaves significantly impact COPD patients, who may experience difficulty breathing.

**Figure:**
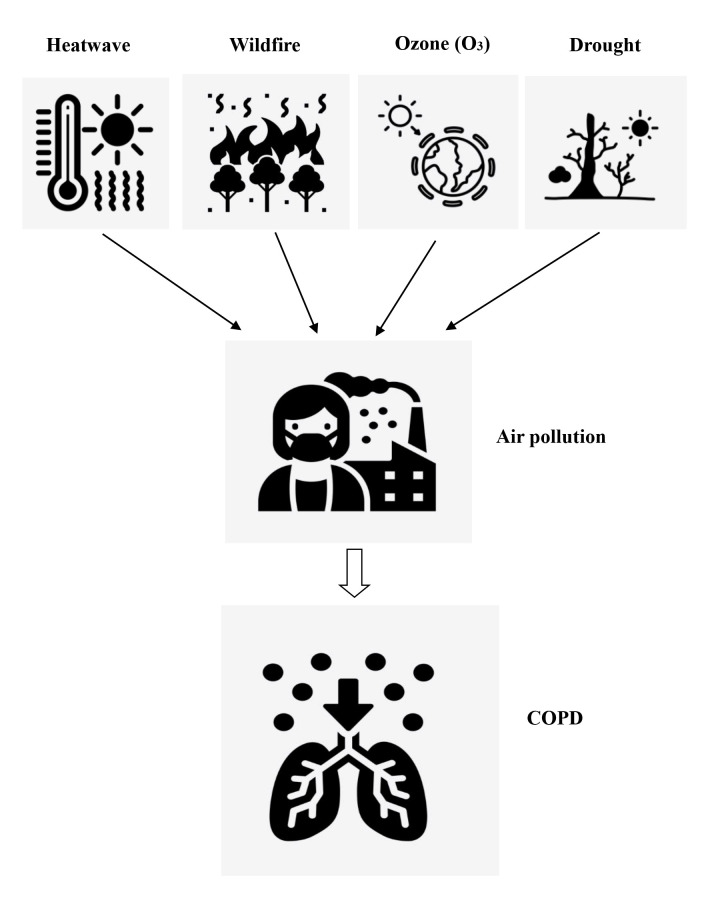
Photo: The relationship between heatwave, wildfire, ozone, drought, and COPD. Extreme weather events such as heatwave, wildfire, ozone pollution, and drought exacerbate air pollution, leading to the worsening of COPD symptoms. Source: authors’ own collection.

Atmospheric circulation anomalies and the formation of multiple high-pressure systems are the direct cause of heatwave formation [14]. Under the control of the warm high-pressure belt, the prevailing downdrafts are conducive to warming the ground. With the role of a wide range of high-pressure belts, the air is drier and less likely to form clouds, making it easier for solar radiation to reach the ground, leading to frequent high temperatures. In addition, when a high-pressure system remains stationary for a long time, a blocking pattern occurs, which disrupts the westerly belt circulation. This creates a prolonged, stable condition where clear, less cloudy weather persists for a considerable period, which can lead to prolonged weather extremes, such as heatwaves [15]. This blocking pattern is most common in the Atlantic and European regions [16].

Global warming and the heat island effect are the main causes of heatwaves. Global warming refers to many greenhouse gases, such as carbon dioxide, methane, nitrous oxide, and ozone, produced by human activities [17]. These greenhouse gases have a high degree of transmittance of visible light coming from the sun’s radiation, and a high degree of absorption of the long-wave radiation reflected from the earth [17]. Greenhouse gases accumulate heat on the earth’s surface and in the atmosphere, increasing temperatures [18]. Under global industrialisation, urbanisation, and population growth, anthropogenic activities such as fossil fuel combustion, deforestation, and industrial production have increased greenhouse gas emissions, increasing the earth’s indicated temperature and global warming. The heat island effect is when temperatures in urban areas are significantly higher than in surrounding suburban or rural areas [19]. With the increase in population and the acceleration of urbanisation, the problem of the urban heat island effect is becoming more and more prominent. Due to the large amount of heat generated by urban buildings, roads, vehicles and other human activities, coupled with the urban surface forests and the lack of vegetation, heat cannot be quickly absorbed and emitted, resulting in increased urban temperatures, exacerbating the urban heat island effect. In the long run, greenhouse gas emissions will increase significantly due to population growth, and the heat island effect will be exacerbated by increased urbanisation. As a result, heatwaves are likely to become more frequent and severe in the future.

The North Atlantic Oscillation and the El Niño-Southern Oscillation have teleconnections with heatwaves. Due to abnormal exchanges and circulation between the atmosphere and the ocean, phenomena such as the North Atlantic and the El Niño-Southern Oscillations occur, leading to changes in regional climate patterns, such as extreme weather events like heatwaves [20,21]. When the Atlantic-European continent North Atlantic Oscillation index is positive in summer, the European continent is prone to high-latitude heatwaves and vice versa for low-latitude heatwaves [15]. The El Niño-Southern Oscillation, generated by the ocean-atmosphere interaction in the equatorial eastern Pacific, leads to anomalously high seawater temperatures in the Pacific Ocean, persistent heavy rainfall in the Americas, and prolonged heatwaves and droughts in Southeast Asia [22].

## RISK OF HEATWAVES FOR COPD PATIENTS

Heatwaves directly exacerbate COPD symptoms through temperature, humidity, and atmospheric pressure. COPD is a respiratory disease sensitive to changes in the surrounding environment, such as temperature, humidity, and air quality. When a heatwave occurs, the human body regulates blood to the surrounding skin tissues to adapt to the heat, dissipating heat through evaporation and other means, resulting in increased blood pressure and faster breathing. At the same time, the low pressure caused by heatwaves reduces the oxygen content in the blood, leading to difficulty breathing. When the airways of COPD patients are exposed to hot gases, cholinergic pathways may be activated, leading to bronchoconstriction in the lungs and triggering dyspnea and coughing [23]. Older adults with respiratory conditions (such as asthma, COPD, and heart disease) are more likely to be at risk for health problems such as coughing, wheezing, and aggravation of respiratory distress during a heatwave. Extremely high temperatures are significantly associated with the risk of death from COPD [24]. For every 1°C increase in temperature, hospitalisation rates for COPD increase by 1.47% [25]. It has been estimated that during the summer months (when temperature ranges from 15.9–40.2°C and the average temperature is 29.5°C), exposure to heatwaves results in a 25% increase in mortality from COPD [26].

Heatwaves cause air pollution and indirectly exacerbate COPD symptoms. Air pollution and high temperatures have a synergistic effect [27]. Droughts and wildfires accompanying heatwaves can exacerbate air pollution, increasing particulate matter (PM) [28]. Air pollution exacerbates cough, asthma and dyspnea symptoms in COPD patients, increasing the risk of hospital admission [29]. A longitudinal cohort study in Taiwan found that long-term exposure to ambient PM with 2.5 microns is also associated with an increased risk of COPD [30]. Heatwaves and droughts lead to less moisture in the air and more sand and dust. Short-term exposure to natural PM with 10 microns during dust storms elevates the risk of hospital admission for COPD exacerbation [31]. In addition, it is worth noting that heatwaves indirectly contribute to ozone pollution. Ozone pollution comes from human activities, especially nitrogen oxides and volatile organic compounds emitted into the atmosphere from vehicle exhaust and factory emissions. Under the strong UV radiation of summer sunlight, these substances absorb the energy of sunlight and undergo photochemical reactions to generate secondary pollutants such as ozone. One recent study in Shanghai found that ozone and heatwaves interact with COPD deaths [24]. This study found that, during the summer heatwave, for every 10 μg/m^3^ increase in ozone, the relative risk associated with COPD deaths was 1.0173 [24].

## IMPACT OF HEATWAVES ON VULNERABLE POPULATIONS, DISEASES AND LOW- AND MIDDLE-INCOME COUNTRIES GLOBALLY

Older people, patients with chronic diseases, and low-income individuals are high-risk groups for heatwaves. The global population of individuals aged ≥65 years is expected to more than double, increasing from 761 million in 2021 to 1.6 billion by 2050 [32]. COPD is one of the most common non-communicable diseases worldwide. It is projected that the number of COPD cases worldwide in individuals aged ≥25 years will rise by 23% between 2020–50, nearing 600 million patients globally by 2050 [33]. It is reported that older people aged >65 years are the primary heatwave victims, and most COPD patients are older people [34]. As the global population ages and the number of COPD patients increases, the adverse effects of heatwaves on health are becoming increasingly prominent. Most research on heatwave deaths comes from high-income countries. The study of deaths from heatwaves in middle- and low-income countries is limited and may be underestimated. In middle- and low-income countries, fragile and limited health care resources may be insufficient to withstand the impact of heatwaves, resulting in more severe illness and fatalities than in high-income countries. Frequent heatwaves pose increasingly severe global health challenges, especially in middle- and low-income countries.

Heatwaves exacerbate the global gap between high-income and low-income countries. This is because heatwaves have a much greater economic impact on low-income countries than high-income ones, further increasing inequality. Callahan and Mankin reported that from 1992 to 2013, cumulative global losses from anthropogenic extreme heat likely ranged between USD 16 trillion and USD 50 trillion [35]. Regions in the lowest income decile experienced annual losses of 8% of gross domestic product per capita, compared to 3.5% in the top income decile. Since most poorer countries are closer to tropical regions, heatwaves cause higher temperatures and greater impacts, resulting in greater losses. It is a tragedy for low-income countries that the countries and regions least responsible for global warming disproportionately bear the cost of heatwaves. Therefore, high-income countries are responsible for raising funds to help low-income countries adapt to climate change.

## HOW TO DEAL WITH HEATWAVES?

First, heatwaves are a global climate challenge that requires countries to collaborate in developing adaptation and mitigation policies under the framework of the United Nations. For example, greenhouse gas emission targets should be adjusted accordingly, with stricter targets set if necessary. Additionally, using clean energy sources, such as wind, hydro, nuclear, and solar, should be encouraged to reduce reliance on fossil fuels like oil and coal, thereby reducing carbon emissions. Increasing subsidies for purchasing electric vehicles and phasing out fuel vehicles are also crucial steps. The faster we transition to a clean energy economy, the more significant the benefits of mitigating climate change will be [36].

Second, improve the collection of national climate data and establish an early warning system. Develop a heatwave vulnerability index based on historical data, the urban heat island effect, and the socio-economic characteristics of the population. Create city risk maps, prepare and rehearse plans based on scenario simulations, and publish public health protection guidelines. Launch an early warning system for high-temperature health risks and an intelligent emergency decision-making platform. Formulate precise interventions based on changes in high-temperature spatial and temporal dynamics, the distribution of public service facilities, and crowd activity patterns. Additionally, establish a platform for information sharing, resource exchange, and coordinated action among government departments of meteorology, health, planning, and emergency response. This platform should integrate and implement heatwave mitigation measures, systematically addressing health risks.

Third, urban comfort space planning should be implemented to reduce the heat island effect. This includes promoting green roofs, cooling coated pavements, and climate-adaptive construction techniques, increasing the coverage of vegetation and water systems, and enhancing the city’s blue-green interwoven ecological space. Also, building height, density, layout form, and volume should be controlled to improve ventilation and heat dissipation. Utilise ecological cold sources such as lakes, rivers, and mountains to build ventilation corridors that introduce fresh, cool, and moist air into urban areas.

Fourth, international cooperation and resource allocation should be strengthened. High-income countries should enhance their support for climate adaptation projects in low-income countries. Developed countries should establish a climate change fund under the framework of the United Nations to provide financial support to low-income countries, helping them adapt to climate change by planting drought-resistant crops, building flood control seawalls, and establishing hurricane warning systems. Additionally, the threshold for applying for funds should be lowered, and the application process should be simplified to enable low-income countries to fully and effectively use these funds to cope with climate change.

## CONCLUSIONS

The United Nations Sustainable Development Goal 13 aims to take urgent action to address climate change and its impacts. While countries strive to combat climate change, the results do not seem satisfactory. Global warming and heatwaves appear to be worsening rather than abating. Extreme weather events pose a serious threat to human health. We highlight the significant threat that heatwaves pose to individuals with chronic respiratory diseases, such as COPD, as well as to older persons and residents of low-income countries. We call on policymakers, health care providers, and individuals to pay more attention to the impacts of heatwaves on human health. We hope to unite global political, economic, and social forces to take the measures outlined above to address the global climate change crisis effectively.
